# Sex bias in prediction and diagnosis of cardiac surgery associated acute kidney injury

**DOI:** 10.1186/s12882-024-03614-x

**Published:** 2024-05-22

**Authors:** Sevag Demirjian, Anne Huml, Faisal Bakaeen, Emilio Poggio, Mariya Geube, Andrew Shaw, A. Marc Gillinov, Crystal A. Gadegbeku

**Affiliations:** 1 Department of Kidney Medicine, Cleveland Clinic, 9500 Euclid Avenue, Q7, Cleveland, OH 44195 USA; 2 Department of Thoracic and Cardiovascular Surgery, Cleveland Clinic, Cleveland, OH USA; 3 Department of Cardiothoracic Anesthesiology, Cleveland Clinic, Cleveland, OH USA; 4 Department of Intensive Care and Resuscitation, Cleveland Clinic, Cleveland, OH USA

**Keywords:** Acute kidney injury, Cardiac surgery, Patient sex, Body surface area, Estimated glomerular filtration rate

## Abstract

**Background:**

Female sex has been recognized as a risk factor for cardiac surgery associated acute kidney injury (CS-AKI). The current study sought to evaluate whether female sex is a risk factor for CS-AKI, or modifies the association of peri-operative change in serum creatinine with CS-AKI.

**Methods:**

Observational study of adult patients undergoing cardiac surgery between 2000 and 2019 in a single U.S. center. The main variable of interest was registered patient sex, identified from electronic medical records. The main outcome was CS-AKI within 2 weeks of surgery.

**Results:**

Of 58526 patients, 19353 (33%) were female; 12934 (22%) incurred AKI based on ≥ 0.3 mg/dL or ≥ 50% rise in serum creatinine (any AKI), 3320 (5.7%) had moderate to severe AKI, and 1018 (1.7%) required dialysis within 2 weeks of surgery. Female sex was associated with higher risk for AKI in models that were based on preoperative serum creatinine (OR, 1.35; 95% CI, 1.29–1.42), and lower risk with the use of estimated glomerular filtration, (OR, 0.90; 95% CI, 0.86–0.95). The risk for moderate to severe CS-AKI for a given immediate peri-operative change in serum creatinine was higher in female compared to male patients (*p* < .0001 and *p *< .0001 for non-linearity), and the association was modified by pre-operative kidney function (*p* < .0001 for interaction).

**Conclusions:**

The association of patient sex with CS-AKI and its direction was dependent on the operational definition of pre-operative kidney function, and differential outcome misclassification due to AKI defined by absolute change in serum creatinine.

**Supplementary Information:**

The online version contains supplementary material available at 10.1186/s12882-024-03614-x.

## Introduction

Female sex has been recognized as a risk factor for cardiac surgery associated acute kidney injury (CS-AKI) as part of several predictive risk models [1, 2]. The Cleveland Clinic model, which has performed well in multiple external cohorts since its inception, assigns a score to female sex on par to comorbidities such as chronic heart failure and insulin requiring diabetes mellitus [1, 3–8]. Subsequently, female sex was included as a risk factor for acute kidney injury in the 2012 *Kidney Disease: Improving Global Outcomes (KDIGO) Clinical Practice Guideline for Acute Kidney Injury (AKI) *[9]*.*

Recent reports, however, contradicted the early observation that posited female sex as a risk factor for AKI. Furthermore, studies based on KDIGO definition of AKI, and administrative data attributed a protective role to female sex [10–12]. Meta-analysis of 28 studies which included close to 7 million patients (2.3 million women) between 2003 and 2018, showed a higher incidence of hospital acquired and non-cardiac surgery associated AKI in men [13]. Experimental animal models implicate testosterone as the mediator of higher risk for AKI in male patients [14–19].

The current study sought to evaluate whether female sex (a) is a risk factor for CS-AKI, (b) modifies the association of peri-operative change in serum creatinine and AKI, and (c) its added value in AKI prediction.

## Methods

### Study setting

The current observational study used data between the years of 2000 and 2019 from a single quaternary, high volume, and referral medical center in United States. Surgical data were obtained from the Anesthesiology Institute Patient Registry and laboratory data were retrieved from electronic medical records. Patient comorbidities and procedures were characterized using the *International Classification of Diseases, Ninth and Tenth Revisions (ICD-9 and ICD-10)*.

The Cleveland Clinic Institutional Review Board provided Ethics approval and waived the need for informed consent due to low risk and observational nature of the study.

Adult patients (≥ 18 years) who underwent coronary artery bypass graft (CABG), valve or aorta surgery were included in the study. Valve surgery included aortic, mitral, pulmonary and tricuspid valve surgery. Aorta surgery included root, ascending and thoraco-abdominal surgery. Patients with end stage kidney failure, received dialysis within six months prior or on day of surgery, or had preoperative serum creatinine of 4 mg/dL or greater were excluded from the study.

### Exposure and covariates

The main variable of interest was registered patient sex, identified from electronic medical records. Study covariates included age, weight, height, serum creatinine and estimated glomerular filtration rate (eGFR) based on the most recent laboratory measurements available prior to surgery, using creatinine-based CKD-EPI equation without race [20]. Unindexed or raw eGFR (eGFR^RAW^) was calculated by multiplication of patient’s eGFR by body surface area (BSA), per Du Bois & Du Bois and division by 1.73 [21]. Peri-operative change in serum creatinine was calculated based on the difference between first serum creatinine following and last serum creatinine preceding surgery.

### Outcomes

AKI was defined by (1) an increase of ≥ 0.3 mg/dL within 48 h, or ≥ 50% rise in serum creatinine (or dialysis) within 2 weeks of surgery, (2) ≥ 50% rise in serum creatinine or dialysis within 2 weeks of surgery, (3) ≥ 100% rise in serum creatinine or dialysis within 2 weeks of surgery (moderate to severe AKI), and (4) provision of dialysis within 2 weeks of surgery (severe AKI). The most recent pre-operative serum creatinine available was used as baseline. All endpoints were measured by the specific time frames mentioned above or earlier in the case of discharge. Patient follow up concluded at end of January 2020.

### Statistical analysis

Descriptive statistics for continuous variables were presented as median (25th-75th percentiles), and categorical variables as count (percent). Univariate comparison between the two sexes was performed using standardized mean difference. The association between AKI and sex with and without adjustments were carried out by logistic regression analyses and presented as odds ratios (OR) with 95% confidence intervals (95% CI). Logistic regression models were constructed to assess the association between AKI and (1) sex, (2) sex and pre-operative serum creatinine, (3) sex, pre-operative serum creatinine, plus height and weight, (4) sex and pre-operative serum creatinine based eGFR, and (5) sex, pre-operative serum creatinine based eGFR^RAW^, height and weight.

Probability of moderate to severe AKI within 2 weeks of surgery was compared between male and female patients using logistic regression, which included interaction terms with patient sex, based on (1) pre-operative serum creatinine, (2) peri-operative change in serum creatinine adjusted for pre-operative serum creatinine. In male patients with eGFR ≥ 60 mL/min/1.73m^2^, clinical characteristics and operative information was compared between those with pre-operative serum creatinine of ≤ 0.6 vs. > 0.6 mg/dL.

Linear regression models were constructed with eGFR as dependent variable, and (1) patient sex, height, and their interaction term, (2) patient sex, weight, and their interaction term, as independent variables. In addition, the dependence of cardio-pulmonary bypass duration on patient height and weight was analyzed in linear regression model, and the coefficient of determination (R^2^) was calculated. R^2^ is the proportion of variation in the dependent variable that is predicted by the model.

A previously published perioperative metabolic panel derived prediction algorithm was used as our base model to examine added value of patient characteristics (age, sex, height, and weight) in AKI prediction [22]. Briefly, the metabolic model uses perioperative change in serum creatinine, and postoperative blood urea nitrogen, serum sodium, potassium, bicarbonate, and albumin from the first metabolic panel after cardiac surgery in multivariable logistic model to predict moderate to severe AKI. The likelihood ratio test was used to assess if the extended model (base model plus patient age, sex, height and weight) compared to the base model alone improved prediction of AKI risk. In the overall cohort and in predetermined subgroups, (1) discrimination of the base and extended models was assessed using concordance (*C*) statistic, (2) calibration-in-the-large was calculated, and (3) bias corrected calibration curves were constructed to illustrate graphically any bias in predicted values (perfectly calibrated predictions are on the 45 degree line of identity).

As sensitivity analysis, dialysis-requiring AKI was used as an alternative to moderate to severe AKI, in assessing the interaction between patient sex and peri-operative change in serum creatinine. All statistical analyses and plotted graphs were performed using SAS Enterprise Guide (SAS, Inc., version 8.2, Cary, NC, USA), and R statistical package (version 4.1.1; www.r-project.org).

## Results

**Fig. 1:**
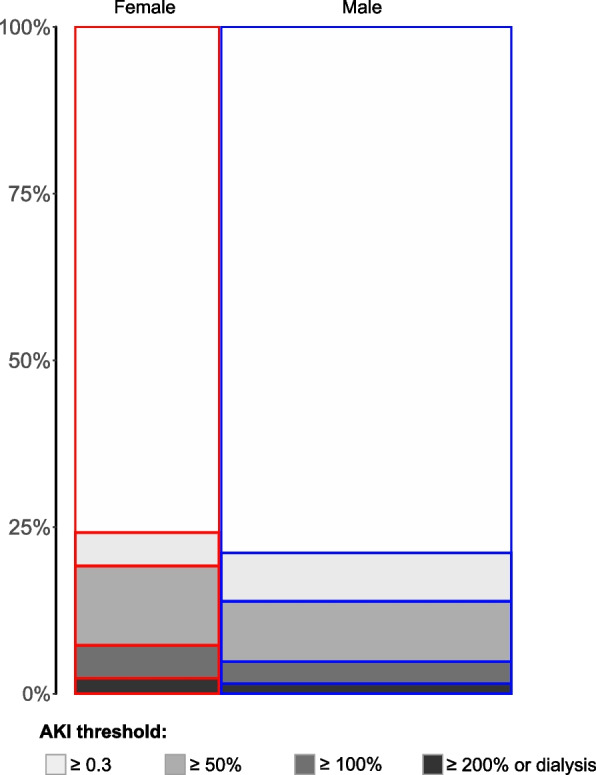
Mosaic plot of AKI incidence per patient sex

Of the 58526 patients analyzed, 19353 (33%) were female; 12934 (22%) incurred AKI based on ≥ 0.3 mg/dL or ≥ 50% rise in serum creatinine (any AKI), 3320 (5.7%) had moderate to severe AKI, and 1018 (1.7%) required dialysis within 2 weeks of surgery. Female sex was associated with older age; 67 (57–75) vs. 65 (56–73) years, shorter stature 160 (157–166) vs. 177 (170–181) cm and smaller body surface area 1.75 (1.62–1.92) vs. 1.97 (1.81–2.13) cm. Female patients had higher incidence of chronic heart failure (28 vs. 20%), and lung disease (17 vs. 11%). Both pre-operative creatinine and eGFR were lower in female patients compared to male counterparts (Table 1). The incidence of any AKI (24 vs 21%, *p* < 0.001), moderate to severe AKI (7.2 vs. 5%, *p* < 0.001) and dialysis requiring AKI (2.3 vs. 1.5%, *p* < 0.001) was higher in female patients (Fig. 1).

**Table 1:** Baseline clinical characteristics, operative information, baseline kidney function and acute kidney injury incidence per participant sex

*Variables*	Female	Male	SMD
	(*n* = 19353)	(*n* = 39173)	
Age, median (IQR), y	67 (57–75)	65 (56–73)	0.11
Race^a^, No. (%)			0.15
Black	1355 (7%)	1410 (4%)	
Non-Black	57171 (93%)	37763 (96%)	
Height (IQR), cm	160 (157–166)	177 (170–181)	1.99
Weight (IQR), kg	70 (60–84)	87 (77–99)	0.84
Body mass index (IQR), kg/m^2^	27 (23–32)	28 (25–31)	0.03
Body surface area (IQR), m^2^	1.75 (1.62–1.92)	1.97 (1.81–2.13)	1.36
Comorbid disease^b^			
Hypertension, No. (%)	12773 (66%)	26246 (67%)	0.02
Diabetes mellitus, No. (%)	4647 (24%)	9011 (23%)	0.04
Congestive heart failure, No. (%)	5421 (28%)	7830 (20%)	0.19
Coronary artery disease, No. (%)	3483 (18%)	9403 (24%)	0.14
Pulmonary disease, No. (%)	3290 (17%)	4309 (11%)	0.17
Pre-surgery eGFR^c^ < 60 mL/min/1.73m^2^, No. (%)	5299 (27%)	7131 (18%)	0.22
Operative procedure^d^			0.07
Valve surgery alone, No. (%)	3292 (17%)	11242 (29%)	
CABG alone, No. (%)	9761 (50%)	13889 (35%)	
Aorta surgery, No. (%)	3383 (17%)	7645 (20%)	
CABG & valve surgery, No. (%)	2916 (15%)	6397 (16%)	
Cardiopulmonary bypass time, min	92 (68–123)	96 (72–125)	0.06
Pre-operative laboratory			
Albumin (IQR), mg/dL	4.1 (3.7–4.4)	4.2 (3.9–4.5)	0.19
Blood urea nitrogen (IQR), mg/dL	18 (14–23)	18 (15–23)	0.03
Pre-surgery serum creatinine (IQR), mg/dL	0.86 (0.71–1.05)	1.04 (0.9–1.2)	0.50
pre-surgery eGFR^c^ (IQR), mL/min/1.73	76 (58–92)	81 (65—94)	0.17
Time to post serum creatinine^e^ (IQR), hr	10.4 (7.3–12.9)	10.3 (7.3–12.6)	0.04
Change in serum creatinine^e^ (IQR), mg/dL	– 0.06 (– 0.14–0.05)	– 0.05 (– 0.15–0.09)	0.08

*Abbreviations*: *IQR* Inter quartile range (25th, 75th), *CABG* Coronary artery bypass graft surgery, *RBC* Red blood cells, *SMD* Standardized mean difference (values > 0.1 are considered significant)

^a^Race information was obtained based on self-identification using fixed categories, retrieved from medical records

^b^Comorbid disease was assessed using the *International Classification of Diseases, Ninth and Tenth Revisions* codes

^c^eGFR calculated by creatinine-based CKD-EPI formula without race

^d^Aorta surgery included root, ascending and thoraco-abdominal aortic surgery. Valve surgery included aortic, mitral, pulmonary and tricuspid valve surgery. ^e^Change from pre-operative serum creatinine based on first measured post surgery

### Pre-operative risk assessment

**Fig. 2:**
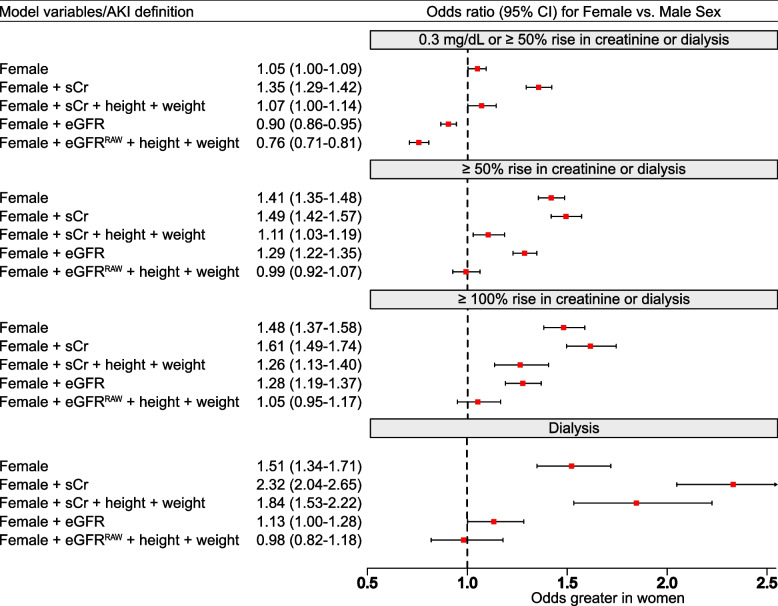
Forest plot of female vs. male odds for AKI incidence by definition used, and method of adjustment for preoperative kidney function. Footnote: eGFR is calculated with pre-operative serum creatinine based on CKD-EPI estimation equation without race. eGFR^RAW^ is calculated by the multiplication of eGFR with patient BSA, and division by 1.73. BSA, stands for body surface area and is calculated per Du Bois & Du Bois method

As depicted in Fig. 2, female sex was associated with higher risk in models that were based on preoperative serum creatinine; the odds ratio ranged from 1.35 (95% CI, 1.29–1.42) when definition included 0.3 mg/dL or more rise in serum creatinine and was highest in patients requiring dialysis (OR, 2.32; 95% CI, 2.04–2.65). Whereas, in models that included eGFR^RAW^, and adjusted for height and weight, female sex was not associated with higher risk. Moreover, if AKI was defined by ≥ 0.3 mg/dL or ≥ 50% increase from baseline, and adjusted for patient eGFR, the risk was lower in female compared to male patients.

Pre-operative serum creatinine based eGFR varied with patient height, however the relationship was steeper in women compared to men (eFigure 1a). The association of eGFR with weight was lower in female vs. male patients across the range studied (eFigure 1b). The R^2^ for cardiopulmonary bypass duration in a multivariable linear regression model that included patient height and weight as independent variables was 0.1%.

**Fig. 3:**
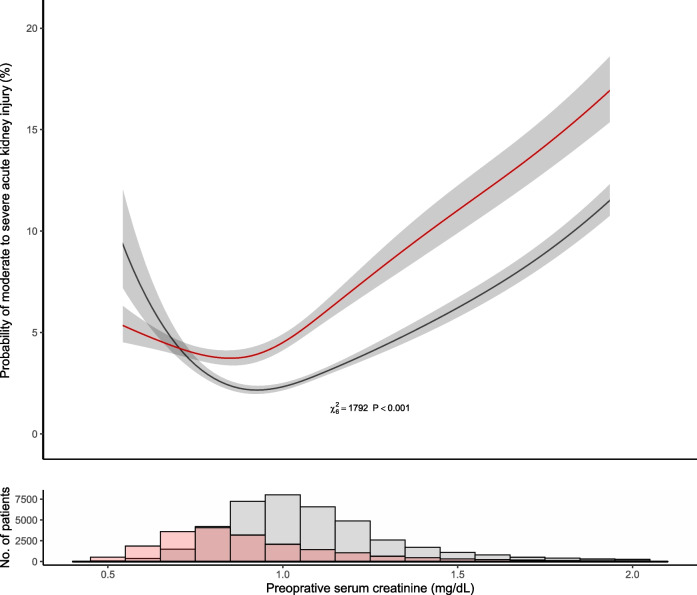
Adjusted probability for moderate to severe acute kidney injury within two weeks of cardiac surgery per pre-operative serum creatinine and patient sex. Footnote: the shaded areas represent 95% confidence interval, based on the multivariable logistic regression model. Accompanying histogram shows female and male patient distribution by pre-operative serum creatinine. Female patients in red and male patients in black

The relationship between pre-operative creatinine and moderate to severe AKI was non-linear (*p *< 0.0001), and the probability of moderate to severe AKI at any creatinine level was dependent on patient’s sex (*p* < 0.0001 for patient sex and serum creatinine interaction term). Female patients had a greater risk for moderate to severe AKI with pre-operative creatinine levels higher than 0.6 mg/dL; whereas the risk of moderate to severe AKI was higher in their male counterparts with lower levels (Fig. 3). There were 466 male patients with pre-operative creatinine of ≤ 0.6 mg/dL, which constituted only 0.8% (466/58526) of the overall cohort, and 1.5% (466/31569) of all male patients with eGFR ≥ 60 ml/min/1.73 m^2^. As summarized in eTable 1, male patients with low pre-operative creatinine compared to patients with high creatinine (and eGFR ≥ 60 ml/min/1.73 m^2^) had shorter stature (175 cm (170–180) vs. 178 cm (171–182)), lower weight (83 kg (70–96) vs. 87 kg (78–99)), and BSA (1.98 m^2^ (1.83, 2.12) vs. 2.05 m^2^ (1.92, 2.19)); these patients also had higher incidence of diabetes mellitus (32% vs. 19%) and chronic lung disease (16% vs. 10%).

### Peri-operative AKI detection

The incidence of AKI based exclusively on ≥ 0.3 mg/dL rise in serum creatinine criterion (without ≥ 50% rise from baseline nor dialysis) was higher in male vs. female patients, 2472 (7.2%) vs. 970 (5%). In the subgroup with pre-operative creatinine > 0.8 mg/dL, AKI due to ≥ 0.3 mg/dL rise in serum creatinine criterion alone was 37% (2642/7152) in male compared to 26% (802/2987) in female patients, *p *< 0.0001. Moreover, due to male preponderance in this group (8 out of 10 patients with pre-operative creatinine > 0.8 mg/dL were male), the number of male patients with AKI solely based on 0.3 rise in serum creatinine was fourfold higher compared to their female counterparts (eFigure 2).

**Fig. 4:**
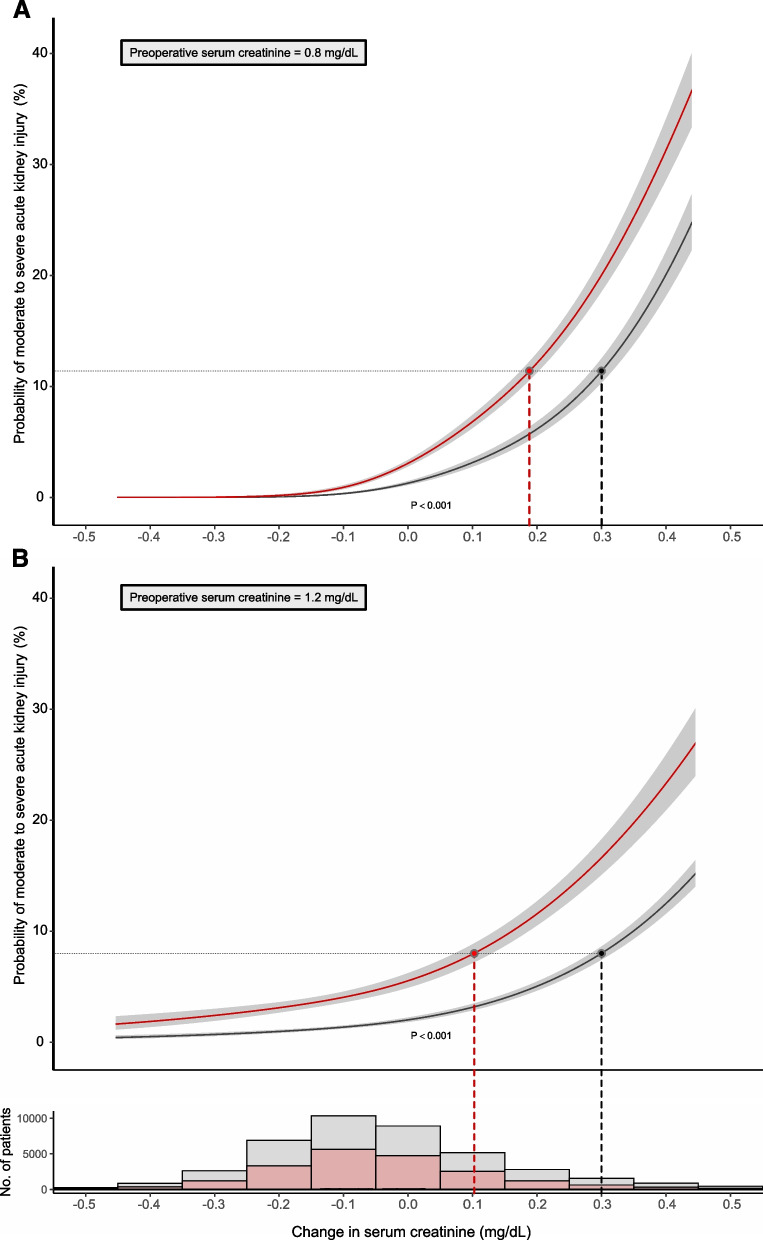
Adjusted probability of acute kidney injury associated with peri-operative change in serum creatinine in male and female patients adjusted for pre-operative serum creatinine of (**a**) 0.8 mg/dL, and (**b**) 1.2 mg/dL. The shaded areas represent 95% confidence interval, based on the multivariable logistic regression model. Accompanying histogram shows female and male patient distribution by peri-operative change in serum creatinine. Female patients in red and male patients in black

Peri-operative change of serum creatinine (based on first blood draw following surgery) was similar in female vs. male patients – 0.06 mg/dL (– 0.14–0.05) vs. – 0.05 (– 0.15, 0.09), and measured at comparable time points post-surgery, Table 1. However, the risk of AKI for a given change in serum creatinine was higher in female patients as shown in Fig. 4 (*p* value < 0.0001 for interaction term). The equivalent risk of AKI in female patients that corresponds to 0.3 mg/dl change of creatinine in male patients, varied based on pre-operative creatinine level (Fig. 4 and eTable 2). For instance, the risk for AKI equivalent to a peri-operative creatinine change of 0.3 mg/dL in male patients, corresponds to (a) change in creatinine of 0.2 mg/dL in female patients when adjusted for pre-operative creatinine of 0.8 mg/dl, and (b) change in creatinine of 0.1 mg/dL when adjusted for pre-operative creatinine of 1.2 mg/dL, (Fig. 4, and eTable 2). In sensitivity analysis, the use of dialysis-requiring AKI as an endpoint showed similar results.

Commensurate with higher probability of moderate to severe AKI with smaller changes in peri-operative creatinine, female sex was also associated with higher values of BUN on post-operative day one, adjusted for pre-operative serum creatinine (eFigure 3).

### Added value in AKI prediction to peri-operative lab-test based model [22]

The log-likelihood ratio test of the base metabolic model compared to the extended model which includes patients’ age, sex, height and weight was significant (*p* < 0.001), indicating that the new variables in the extended model add significant diagnostic value to the base model. The relative explained variation of the extended model relative to the base model was 9%. The AUC of the base metabolic model was 0.854 (95%CI, 0.850–0.861), and 0.866 (95%CI, 0.859–0.872) in the combined model with a significant difference of 0.01 (95% CI, 0.006–0.011).

Calibration-in-the-large, which refers to the agreement between observed and predicted incidence, improved from 5.7% (95% CI, 5.6–5.8) to 7.2% (95% CI, 7.0–7.4) probability risk for moderate to severe AKI, in comparison to observed incidence of 7.2% in female patients. High resolution calibration curves for the base and extended models are displayed in eFigure 4. The extended model improved discrimination and calibration in patient categories with extremes of muscle mass (advanced age, low BSA, and female sex); as seen in eTable3 and eFigure4.

## Discussion

In the current study, both AKI diagnosis and prediction varied in male vs. female patients based on the methods used to estimate baseline kidney function, and criteria used to define AKI (percent vs. absolute change in serum creatinine). The following observations were made: (1) the use of pre-operative serum creatinine as a surrogate for kidney function resulted in higher risk for CS-AKI in female patients due to lack of adjustment for non-GFR determinants of serum creatinine. The risk of CS-AKI was abrogated when eGFR was used to account for sex-based differences in serum creatinine levels due to differences in body muscle mass and creatinine production. (2) At any given absolute change in serum creatinine immediately following surgery, the risk of progression to moderate or severe AKI was higher in female patients compared to an equivalent change in male patients. (3) The use of absolute change in serum creatinine, resulted in higher incidence of 0.3 mg/dL based AKI rates in male vs female patients. (4) The inclusion of patient age, sex, weight and height significantly improved the discrimination and calibration of a previously developed and validated peri-operative serum creatinine-based AKI predictive model in females.

Higher incidence of CS-AKI in female patients has been attributed to higher disease burden due to referral bias [2, 23–25]. In the current study, however, surgery type or cardiopulmonary bypass duration was comparable between the sexes. Preoperative eGFR on the other hand was significantly lower in female patients despite corresponding lower serum creatinine levels.

The discord in estimation of baseline kidney function between serum creatinine and estimated GFR is due to confounding where lower muscle mass overestimates kidney function when based on serum creatinine levels in female vs. male patients. Subsequently predictive models which include baseline serum creatinine will invariably have female gender associated with higher incidence of AKI [2, 23, 26]. In the current cohort, there was a significant interaction between pre-operative serum creatinine and patient sex, where the risk of AKI based on matching serum creatinine was higher in female patients as a consequent of lower corresponding kidney function. In addition, the substitution of creatinine with estimated GFR (adjusted for anthropometric differences) as a surrogate for kidney function, also eliminated the increased AKI risk attributed to female sex [27–29]. Indeed, consistent with the current findings, AKI models studied post cardiac surgery that are based on GFR estimates rather than serum creatinine, did not show female sex as a risk factor [3, 30].

Of note, when eGFR (BSA indexed eGFR) was used to estimate kidney function, there was a significant attenuation of AKI risk associated with female sex. However, this risk was further reduced when eGFR^RAW^was used but with height and weight adjustments performed in a regression model. There are two possible explanations for the additional offset noted in the latter strategy compared to eGFR use. First, there are unaccounted sex differences in BSA estimation that are not captured with the use of equation by Du Bois & Du Bois (irretrievably factored in CKD-EPI equation) [21]. The BSA equation by Du Bois was derived from only 9 subjects, using unsophisticated statistical methods that were available in 1916 [31]. In the present cohort, eGFR showed a differential relationship with height depending on patient’s sex; it showed positive linear correlation in women and no correlation in men. In other words, the relationship with height is not corrected in women as it is in men, by indexing to BSA, which may result in overestimation of GFR in tall and underestimation in short female patients. Moreover, the practice of indexing physiologic measures (such as GFR, cardiac output, etc.), although entrenched in medicine and medical research, is problematic because they rely on strict assumptions which are often violated [32, 33]. In contrast to indexing method, the use of regression to adjust for anthropometric characteristics does not require any assumptions such as having a positive linear relationship, and an intercept of zero. Second, both smaller body size and female sex have been independently associated with smaller coronary arteries; therefore anthropometric measures by their own right could be associated with AKI (rather than through kidney function) [34–36]. In the current study, however, the effect of patient’s height or weight on the duration of cardiopulmonary bypass was negligible, discounting an immediate effect of patient size on surgical outcomes.

In contrast to reports of elevated AKI risk post cardiac surgery, female sex has been deemed protective in other settings [11, 12, 37]. Analysis of Veterans Affairs data, which included about 6 thousand female patients, male sex was identified as a risk factor for post-operative AKI in the fully adjusted model [37]. Similarly, the analysis of large national compilation of surgical outcome data from 121 US hospitals showed male sex to be independently associated with post-operative AKI [38]. In many of these reports, AKI diagnosis was based on absolute (0.3 mg/dL) change in serum creatinine after its incorporation in AKI diagnosis and classification criteria in 2007 [9, 39]. Absolute change in creatinine has been adopted initially based on its association with short term mortality in post cardiac surgery patients [40]. Also time to diagnosis based on absolute change in serum creatinine is considerably shorter compared to percentage based criteria in male patients who on average have higher serum creatinine than female patients; where the higher the pre-operative creatinine the longer it would take to reach percentage based endpoint (such as doubling) due to constant rate of creatinine production [22, 41]. An implicit assumption in the use of absolute changes of creatinine in AKI diagnosis is that an equivalent change in serum creatinine represents the same risk in all patients. However, the higher serum creatinine levels in male patients (caused by larger muscle mass) leads to higher incidence of AKI diagnoses exclusively based on small absolute changes in creatinine. Conversely, due to lower kidney function in female compared to male individuals with an identical serum creatinine, an equivalent absolute change in serum creatinine is much more likely to be clinically consequential in female patients. In our study, there was disproportionately higher number of male patients who were labeled with AKI when absolute change in serum creatinine was used, compared to thresholds based on percent change in serum creatinine or provision of dialysis. Also for the same perioperative change in serum creatinine, female patients were much more likely to progress to moderate to severe AKI, particularly in those with elevated preoperative serum creatinine. Differential misclassification of AKI based on absolute change (or thresholds) in serum creatinine is not restricted to female patients who are historically underrepresented in clinical studies, but also betides children and individuals with low muscle mass (chronic illness, prolonged hospitalization, etc.).

It is also important to consider observed biological and analytical variability in serum creatinine measurements that might affect diagnostic accuracy based on criteria used to define AKI. For example, criteria based on absolute change in serum creatinine increase false positive rates in patients with elevated baseline, such as in male compared to female patients [42]. Whereas, AKI definitions based on percent change in patients with very low baseline serum creatinine (as seen in pediatric population) will be due to analytic or biologic variation rather than change in kidney function [43].

The addition of female sex and other determinants of muscle mass such as age, height and weight to a previously developed and validated peri-operative test based predictive model for CS-AKI, improved model performance in female patients, and other subgroups with low muscle mass [22]. The inclusion of patient sex in the model however should not be construed as an intrinsic biological risk for AKI but as necessary effect modifier when serum creatinine is used as a surrogate for kidney function. In fact, the omission of patient sex (and anthropomorphic measures) will lead to poor model performance at its best and to biased results at its worst in marginalized groups.

Our study has limitations. First, the current results apply to AKI in cardiac surgery setting where the etiology and management strategies of AKI are more homogeneous compared to other settings. This favors the detection of biologic differences, but not other factors related to gender, socioeconomic status or delivery of care. Second, the current study reflects the experience and the processes of care delivered in a large volume cardiac surgery referral center and may not be representative of other practices. Third, baseline kidney function assessment by pre-operative serum creatinine or serum creatinine based eGFR assumes steady state, which may not be applicable to all patients. Fourth, the current study does not directly measure muscle mass or GFR. Fifth, the current study did not evaluate the effect of patient sex on AKI diagnosis and classification based on urine output criteria. Finally, due to the observational nature of the study, the current findings are associations and should be considered hypothesis generating.

In conclusion, although clinical and experimental science point to sex differences in susceptibility to AKI, the current study findings show that the association and its direction was dependent on the operational definition of pre-operative kidney function (overestimation), and differential outcome misclassification due to AKI defined by absolute change in serum creatinine (underestimation). It is important to recognize the implications of methods used in baseline kidney function estimation and AKI outcome classification in vulnerable groups, let alone half the population. Furthermore, with the advent of artificial intelligence and big data analytics there is a risk of magnifying and perpetuating conflated variations that may result in unintended consequences and outcomes.

### Supplementary Information

Supplementary Material 1.

## Data Availability

The authors will review and consider data requests on case by case basis. For inquiries please contact Dr. Sevag Demirjian.
