# Supplementary material for: Sex bias in prediction and diagnosis of cardiac surgery associated acute kidney injury
Source: BMC Nephrol. 2024 May 22;25:180. doi: 10.1186/s12882-024-03614-x (PMC11112848; doi:10.1186/s12882-024-03614-x)
Supplement: Supplementary file 1 — Supplementary Material 1. [file 12882_2024_3614_MOESM1_ESM.docx]

**Supplement 1**

Demirjian et al. Sex Bias in Prediction and Diagnosis of Cardiac Surgery Associated Acute Kidney Injury.

**eTable 1.** Baseline Clinical Characteristics, Operative Information, Baseline Kidney Function and Acute Kidney Injury Incidence per Pre-operative Serum Creatinine Among Male Participants with estimated GFR ≥ 60 ml/min/1.73 m^2^.

**eTable 2.** Creatinine-adjusted Probability of Moderate to Severe Acute Kidney Injury within 2 Weeks by Patient Sex.

**eTable 3.** Calibration-in-the-large, and discrimination of the base^a^ and extended^b^ models in predicting AKI within 2 weeks of surgery.

**eFigure 1a.** Association of Height with Pre-operative Serum Creatinine Based eGFR^BSA^ per Patient Sex.

**eFigure 1b.** Association of Weight with Pre-operative Serum Creatinine Based eGFR^BSA^ per Patient Sex.

**eFigure 2.** Mosaic Plot of AKI Incidence per Patient Sex and Pre-operative Serum Creatinine Level.

**eFigure 3.** Boxplots of Blood Urea Nitrogen Levels per Pre-operative Serum Creatinine, and Peri-operative Change in Serum Creatinine Between Female and Male Patients.

**eFigure 4a.** Calibration Plot of Moderate to Severe Acute Kidney Injury within Two Weeks of Surgery in the Overall Cohort. Vertical Axis Represents Observed Incidence, and Horizontal Axis the Predicted Probability. Histogram Shows Patient Distribution in a Logarithmic Scale across the Predicted Probability.

**eFigure 4b.** Calibration Plot of Moderate to Severe Acute Kidney Injury within Two Weeks of Surgery in Patients 80 years and older. Vertical Axis Represents Observed Incidence, and Horizontal Axis the Predicted Probability. Histogram Shows Patient Distribution in a Logarithmic Scale across the Predicted Probability (overall cohort in light grey; patients ≥ 80 y.o. in dark grey).

**eFigure 4c.** Calibration Plot of Moderate to Severe Acute Kidney Injury within Two Weeks of Surgery in Patients with BSA ≤ 1.6m^2^. Vertical Axis Represents Observed Incidence, and Horizontal Axis the Predicted Probability. Histogram Shows Patient Distribution in a Logarithmic Scale across the Predicted Probability (overall cohort in light grey; patients with BSA ≤ 1.6m^2^ in dark grey).

**eFigure 4d.** Calibration Plot of Moderate to Severe Acute Kidney Injury within Two Weeks of Surgery in Female Patients. Vertical Axis Represents Observed Incidence, and Horizontal Axis the Predicted Probability. Histogram Shows Patient Distribution in a Logarithmic Scale across the Predicted Probability (overall cohort in light grey; female patients in dark grey).

| eTable 1. Baseline Clinical Characteristics, Operative Information, Baseline Kidney Function and Acute Kidney Injury Incidence per Pre-operative Serum Creatinine Among Male Participants with estimated GFR ≥ 60 ml/min/1.73 m^2^. | | | |
| --- | --- | --- | --- |
| *Variables* | sCr ≤ 0.6 mg/dL | sCr > 0.6 mg/dL | SMD |
|  | (n=466) | (n=31569) |  |
| Age, median (IQR), y | 58 (50-69) | 62 (54-70) | 0.25 |
| Race^a^, No. (%) |  |  | 0.20 |
| Black | 11 (2%) | 1138 (4%) |  |
| non-Black | 455 (98%) | 30431 (96%) |  |
| Height (IQR), cm | 175 (170-180) | 178 (171-182) | 0.25 |
| Weight (IQR), kg | 83 (70-96) | 87 (78-99) | 0.28 |
| Body mass index (IQR), kg/m^2^ | 27 (23-31) | 28 (25-31) | 0.16 |
| Body surface area (IQR), m^2^ | 1.98 (1.83-2.12) | 2.05 (1.92-2.19) | 0.33 |
| Comorbid disease^b^ |  |  |  |
| hypertension, No. (%) | 301 (65%) | 19954 (63%) | 0.03 |
| diabetes mellitus, No. (%) | 147 (32%) | 6154 (19%) | 0.28 |
| congestive heart failure, No. (%) | 86 (18%) | 4875 (15%) | 0.08 |
| coronary artery disease, No. (%) | 108 (23%) | 6854 (22%) | 0.03 |
| pulmonary disease, No. (%) | 74 (16%) | 3138 (10%) | 0.18 |
| Operative procedure^c^ |  |  | 0.25 |
| valve surgery alone, No. (%) | 175 (38%) | 9138 (29%) |  |
| CABG alone, No. (%) | 129 (28%) | 11839 (38%) |  |
| aorta surgery, No. (%) | 65 (14%) | 5199 (16%) |  |
| CABG & valve surgery, No. (%) | 97 (21%) | 5393 (17%) |  |
| Cardiopulmonary bypass time, min | 94 (70-124) | 94 (70-122) | 0.04 |
| Pre-operative laboratory |  |  |  |
| albumin (IQR), mg/dL | 4 (3.4-4.3) | 4.3 (4.0-4.5) | 0.62 |
| blood urea nitrogen (IQR), mg/dL | 13 (10-16) | 17 (14-20) | 0.85 |
| serum creatinine (IQR), mg/dL | 0.60 (0.58-0.62) | 0.99 (0.89-1.09) | 3.65 |
| eGFR (IQR), mL/min/1.73 m^2^ | 111 (103-120) | 82 (72-92) | 2.11 |
| Time to post serum creatinine (IQR), hr | 9.5 (6.2-12.6) | 10.5 (7.4-12.8) | 0.20 |
| Change in serum creatinine (IQR), mg/dL | 0.05 (– 0.02-0.14) | – 0.05 (– 0.13-0.07) | 0.57 |
| Abbreviations: IQR, inter quartile range (25^th^, 75^th^); CABG, coronary artery bypass graft surgery; SMD, standardized mean difference (values > 0.1 are considered significant). ^a^Race information was obtained based on self-identification using fixed categories, retrieved from medical records. ^b^Comorbid disease was assessed using the *International Classification of Diseases, Ninth and Tenth Revisions* codes*.* ^c^Aorta surgery included root, ascending and thoraco-abdominal aortic surgery. Valve surgery included aortic, mitral, pulmonary and tricuspid valve surgery. | | | |

| eTable 2. Creatinine-adjusted Probability of Moderate to Severe Acute Kidney Injury within 2 Weeks by Patient Sex. | | | | | | | | | |
| --- | --- | --- | --- | --- | --- | --- | --- | --- | --- |
|  | Probability of moderate to severe AKI (%) vs. *Δ*Cr=0.3 mg/dL in Male Patients | | | | | | | | |
|  |  | Male (Ref.) |  |  |  | Female |  |  |  |
| Pre-operative |  | *Δ*Cr=0.3 |  | *Δ*Cr=0.1 | *Δ*Cr=0.15 | *Δ*Cr=0.2 | *Δ*Cr=0.25 | *Δ*Cr=0.3 |  |
| Cr = 0.4 |  | 37.4 (30.6-44.6) |  | 10.7 (8.8-13.0) | 15.5 (13.0-18.5) | 21.2 (17.9-24.9) | 28.1 (23.8-32.9) | 36.2 (30.6-42.2) |  |
| Cr = 0.6 |  | 23.6 (20.3-27.3) |  | 9.3 (8.2-10.6) | 12.9 (11.5-14.4) | 17.1 (15.3-19.0) | 22.2 (19.9-24.7) | 28.4 (25.3-31.7) |  |
| Cr = 0.8 |  | 13.9 (12.8-15.1) |  | 8.1 (7.5-8.8) | 10.6 (9.9-11.4) | 13.6 (12.8-14.6) | 17.3 (16.2-18.5) | 21.7 (20.2-23.3) |  |
| Cr = 1.0 |  | 9.1 (8.6- 9.7) |  | 7.7 (7.0-8.4) | 9.6 (8.8-10.3) | 11.8 (10.9- 12.8) | 14.5 (13.4-15.7) | 17.8 (16.4-19.3) |  |
| Cr = 1.2 |  | 8.8 (8.2-9.5) |  | 8.8 (8.0-9.7) | 10.6 (9.6-11.6) | 12.7 (11.6-13.8) | 15.2 (13.9-16.6) | 18.0 (16.5-20.0) |  |
| Cr = 1.6 |  | 14.4 (13.5-15.3) |  | 14.9 (13.5-16.4) | 17.0 (15.5-18.6) | 19.4 (17.8-21.2) | 22.1 (20.3-24.1) | 25.1 (23.0-27.3) |  |
| Cr = 2.0 |  | 23.5 (21.9-25.3) |  | 24.6 (21.8-27.7) | 26.9 (23.9-30.0) | 29.3 (26.2-32.6) | 31.9 (28.6-35.4) | 34.6 (31.0-38.3) |  |
| Abbreviations: Cr, serum creatinine; *Δ*Cr, peri-operative change in serum creatinine (last measured pre-surgery minus first measure post-surgery). *Probability in the reference group (*Δ*Cr=0.3 mg/dL in male participants). Red shade denotes change in creatinine predicted in female participants which correspond to male counterparts with 0.3 mg/dL perioperative change in serum creatinine (grey shade); extent of confidence interval overlap equivalent to p > .05. | | | | | | | | | |

| eTable 3. Calibration-in-the-large, and discrimination of the base^a^ and extended^b^ models in predicting AKI within 2 weeks of surgery. | | | | | | |
| --- | --- | --- | --- | --- | --- | --- |
|  |  | Calibration-in-the-large | | C-statistic | | |
|  | Observed | Base^a^ | Extended^b^ | Base^a^ | Extended^b^ | Difference |
| All patients | 5.7 | 5.7 (5.6-5.8) | 5.7 (5.6-5.8) | 0.854 (0.850-0.861) | 0.866 (0.859-0.872) | + 0.012 (0.007-0.013) |
| Age ≥ 80 | 7.6 | 6.2 (6.0-6.5) | 7.6 (7.4-7.9) | 0.801 (0.780-0.822) | 0.814 (0.793-0.834) | + 0.013 (0.004-0.018) |
| Female sex | 7.2 | 5.8 (5.6-5.9) | 7.2 (7.0-7.4) | 0.832 (0.821-0.843) | 0.839 (0.828-0.850) | + 0.007 (0.002-0.009) |
| BSA^c^ ≤ 1.6 | 7.1 | 5.8 (5.5-6.1) | 7.0 (6.7-7.4) | 0.805 (0.778-0.831) | 0.812 (0.787-0.837) | + 0.007 (– 0.016-0.001) |
| AKI, acute kidney injury; BSA, body surface area.  ^a^Peri-operative test-based AKI predictive model{Demirjian, 2022 #4890}. ^b^Extended model includes the base model plus age, gender, height and weight. ^c^Mean predicted percentiles with 95% confidence interval. ^c^BSA calculated based on DuBois and DuBois formula (0.007184 × Weight^0.425^ × Height^0.725^). {Du Bois, 1989 #5262} | | | | | | |

eFigure 1a. Association of Height with Pre-operative Serum Creatinine Based eGFR per Patient Sex.


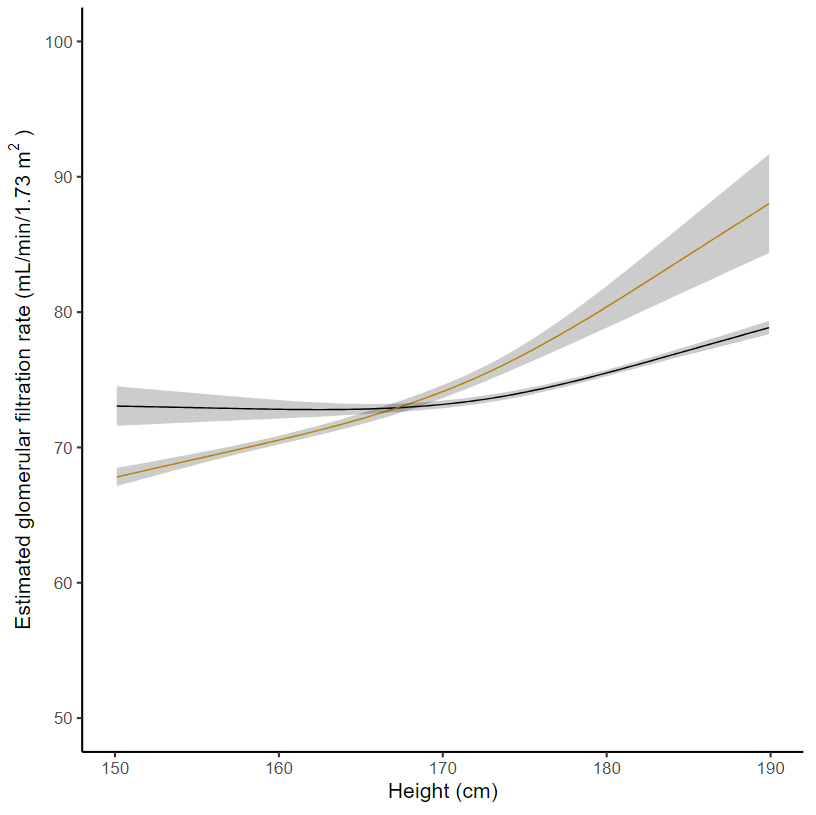


eFigure1b. Association of Weight with Pre-operative Serum Creatinine Based eGFR per Patient Sex.


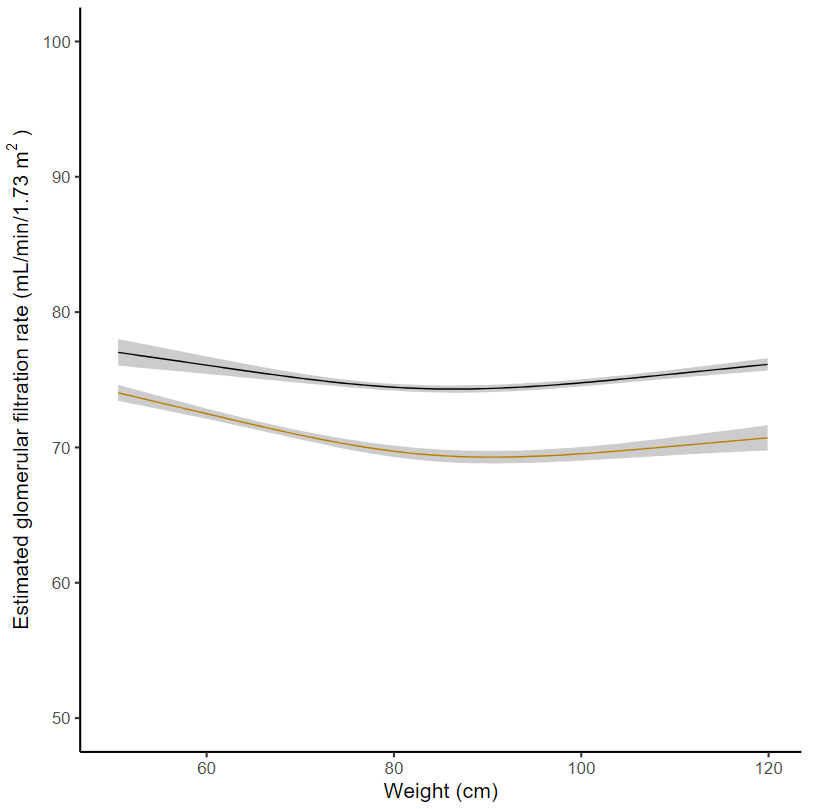


eFigure 2. Mosaic Plot of AKI Incidence per Patient Sex and Pre-operative Serum Creatinine Level. Sum of all percentages shown within blocks is 100% (Percentile < 0.3% not shown).

eFigure 3. Boxplots of Blood Urea Nitrogen Levels per Pre-operative Serum Creatinine, and Peri-operative Change in Serum Creatinine between Female and Male Patients.

eFigure 4a. Calibration Plot of Moderate to Severe Acute Kidney Injury within Two Weeks of Surgery in the Overall Cohort. Vertical Axis Represents Observed Incidence, and Horizontal Axis the Predicted Probability. Histogram Shows Patient Distribution in a Logarithmic Scale across the Predicted Probability.

eFigure 4b. Calibration Plot of Moderate to Severe Acute Kidney Injury within Two Weeks of Surgery in Patients 80 years and older. Vertical Axis Represents Observed Incidence, and Horizontal Axis the Predicted Probability. Histogram Shows Patient Distribution in a Logarithmic Scale across the Predicted Probability (overall cohort in light grey; patients ≥ 80 y.o. in dark grey).

eFigure 4c. Calibration Plot of Moderate to Severe Acute Kidney Injury within Two Weeks of Surgery in Patients with BSA ≤ 1.6m^2^. Vertical Axis Represents Observed Incidence, and Horizontal Axis the Predicted Probability. Histogram Shows Patient Distribution in a Logarithmic Scale across the Predicted Probability (overall cohort in light grey; patients with BSA ≤ 1.6m^2^ in dark grey).

eFigure 4d. Calibration Plot of Moderate to Severe Acute Kidney Injury within Two Weeks of Surgery in Female Patients. Vertical Axis Represents Observed Incidence, and Horizontal Axis the Predicted Probability. Histogram Shows Patient Distribution in a Logarithmic Scale across the Predicted Probability (overall cohort in light grey; female patients in dark grey).
